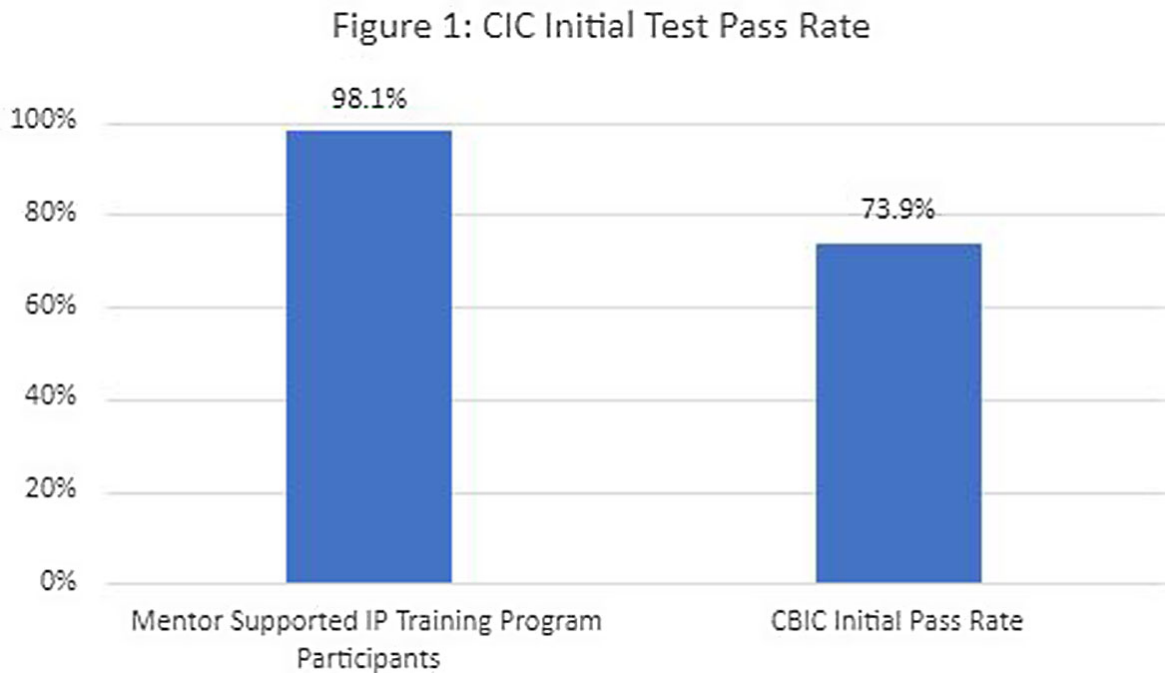# Bridging the Gap: Specialized Training Programs for Infection Prevention Specialists Increase Certification Success

**DOI:** 10.1017/ash.2024.258

**Published:** 2024-09-16

**Authors:** Kelly Holmes, Jennifer McCarty, Sandi Steinfeld, Kelley Boston

**Affiliations:** Infection Prevention & Management Associates

## Abstract

**Background:** The role of the infection preventionist (IP) is complex and encompasses a range of responsibilities requiring extensive knowledge in infection control practices, data analysis, surveillance, performance improvement and collaboration with multidisciplinary teams. Infection prevention certification (CIC) by the certification Board of Infection Control (CBIC) is a standardized marker of knowledge and competencies required for practice in the field. In a 2020 survey of IPs, less than half were certified or planned to become certified. Of those that do take the certification exam, less than three quarters pass on their initial exam attempt. **Methods:** From 2017 to 2023, fifty-two new IPs were enrolled in a competency-based training program which combined didactic and applied learning on core IP job functions, and a structured mentoring program. The initial didactic phase consisted of evidence-based learning modules with validation of competency through post-training testing and practical demonstration. Education was provided by an advanced practice IP via remote webinars, which included discussion of questions, skills coaching, and review of post-tests. Novice IPs were partnered with at least two preceptors: one advanced practice lead preceptor guided the novice IPs through assigned education modules and oversaw program management and training benchmarks. A second, near-peer preceptor or mentor collaborated with the novice IP in the facility setting. Initial training focused on facility operations, surveillance, rounding and other facility specific activities. Facility mentors were responsible for combining education module topics with practical application of skills. Mentors guided novice IPs through National Healthcare Surveillance network (NHSN) surveillance training and validated surveillance and infection coding until the novice IP had an interrater reliability validation assessing surveillance competency. After the initial training phase, the novice IPs began preparation for certification. This phase included additional training modules aligned with the CBIC certification content outline and practice exams. **Results:** All 52 novice IPs completed the training program and attempted the CIC examination. The initial pass rate for the certification exam among IPs in the supervised training and mentorship program was 98.1% (n=51). This is 33% higher than the initial pass rate published by CBIC, which was 73.9% (Figure 1). **Conclusions:** Organizing evidence-based guidelines into topic-specific modules builds a foundation of infection prevention and control knowledge, which is enhanced through remote instruction and direct application of skills under a preceptor’s supervision. This method allows IPs to be introduced to concepts covered in the board certification exam upon hire and support certification with improved outcome

**Figure:**